# Disciplinary Imbalances in Urology and Gynecology Research Publications within Functional Urology

**DOI:** 10.3390/clinpract14050139

**Published:** 2024-08-29

**Authors:** Sümeyye Kozan, Mohammad Sajjad Rahnamai, Jasmin Ataei, Janina Dombrowski, Laila Najjari

**Affiliations:** Department of Gynaecology and Obstetrics, University Hospital RWTH Aachen, 52074 Aachen, Germany

**Keywords:** gynecology, functional urology, research publications

## Abstract

(1) Background: This study aimed to quantify and evaluate the publication rate and discrepancies of functional urology abstracts from international conferences, and to explore the interdisciplinary contributions of urology and gynecology to the field. (2) Methods: A retrospective bibliometric and content analysis was conducted on abstracts presented between 2015 and 2019 at the EAU and ICS congresses, focusing on functional-urological keywords. A discrepancy scoring system ranging from 0 (minor discrepancies) to 3 (significant discrepancies) assessed the consistency between conference abstracts and full-text publications, and an in-depth analysis determined the disciplinary origin of these publications. (3) Results: Between 2015 and 2019, 53% of EAU and 57% of ICS congress abstracts were published as full-text articles, with minor discrepancies in 38% of EAU and 49% of ICS publications, and significant discrepancies in 17% from both. Urology departments dominated publications, contributing 68% at EAU and 55% at ICS, whereas gynecology contributed only 1% at EAU and 12% at ICS. (4) Conclusions: This study illuminates the need for improved reporting standards and interdisciplinary collaboration in functional urology, as well as increased gynecology research in functional urology-related fields, suggesting that addressing these issues is crucial for advancing the field and enhancing patient care.

## 1. Introduction

Functional urology addresses conditions affecting the lower urinary tract and pelvic floor, such as urinary incontinence, pelvic organ prolapse, and overactive bladder, significantly impacting global health and patient well-being [1,2]. With an increased incidence in aging populations, especially among women, these disorders necessitate targeted research and interventions [3,4,5]. Moreover, the field explores innovative treatments and surgical options for conditions like stress urinary incontinence, psychogenic urinary retention, and interstitial cystitis/bladder pain syndrome, alongside advancements in tissue engineering for urinary tract reconstruction [6,7,8,9].

In the clinical domain, urologists and urogynecologists, the latter specializing in female pelvic medicine and reconstructive surgery, lead in managing these conditions. Urogynecologists are particularly focused on surgical and non-surgical interventions for pelvic floor disorders, underscoring the evolution of specialized care within this field [10,11,12]. Furthermore, gynecologists play a significant role in addressing urological conditions associated with gynecological health, especially urinary incontinence and pelvic floor dysfunction post-childbirth [13,14].

Interdisciplinary collaboration among urologists, urogynecologists, gynecologists, and professionals from adjacent specialties such as oncology and physiotherapy, is fundamental in the comprehensive management of functional urological disorders. This collaborative model facilitates the integration of diverse expertise and perspectives, crucial for the development of innovative treatment modalities and the enhancement of patient care protocols [15,16].

The integrity of scientific communication, particularly in medical fields, is pivotal for the progression of medical knowledge and enhancement of patient care, emphasizing the need for consistency between conference abstracts and their subsequent full-text publications. Some researchers emphasize the need for a systematic framework and effective communication to uphold research integrity, while others highlight the crucial role of authors and editors in preserving the integrity of scientific communication [17,18,19,20]. This consistency ensures the accurate representation of initial findings, facilitating reliable and effective clinical guidelines and patient care strategies [21,22].

A range of studies have highlighted the potential for discrepancies between abstracts and full reports to distort perceptions of research validity and applicability. Li (2017) found that abstracts in primary biomedical research are frequently inconsistent with full reports, with a median inconsistency level of 39% [23]. This inconsistency can be influenced by factors such as the time interval between the two versions [23]. Jarvis (2016) and Shakiba (2014) both underscore the need for enhanced transparency and consistency in reporting, with Jarvis specifically noting the impact on public health [24,25]. Falagas (2006) and Costa (2022) further emphasize the potential impact on clinical decision-making and public acceptance of research findings [26,27].

There exists a notable gap in the literature concerning a detailed, discipline-specific analysis of contributions and roles of urology and gynecology within functional urology in advancing research and clinical practice within this field. This gap points to a need for a more detailed exploration of how different medical disciplines contribute to the evolving narrative of functional urology. By addressing this need, our study aims to fill this void, providing a nuanced understanding of the dynamics between various specialties.

Thus, the aim of this study was to quantify the proportion of functional urology abstracts presented at both the European Association of Urology (EAU) and the International Continence Society (ICS) Congress, which serve as prominent platforms for disseminating latest research and clinical practices in urology and continence care, that were subsequently published as full scientific papers. We sought to analyze these abstracts using a bespoke discrepancy index to assess and quantify any differences between the conclusions presented at these congresses and those outlined in the final publications. Additionally, the study aimed to examine the disciplinary origins of these functional urology-related publications from these conferences, thereby evaluating the contribution of different medical specialties to the field of functional urology.

## 2. Materials and Methods

This was a retrospective bibliometric analysis combined with an element of content analysis, with the core of this study involving examining the publication patterns of scientific abstracts, including their presence in conference proceedings and subsequent publication in scientific journals. Bibliometric analyses are used to explore the quantitative aspects of academic literature, such as publication rates, authorship patterns, and the distribution of topics across journals and conferences [28,29,30]. Content analysis in this context involves the systematic coding and categorizing of text (in this case, the abstracts) to quantify specific elements, such as the extent of deviation between versions of the abstracts.

For this study, all abstracts presented between 2015 and 2019 at the congresses of the EAU and ICS were selected by two independent reviewers (SK, SMR) based on specific functional-urological keywords, including Benign Prostatic Hyperplasia (BPH), Bladder Outlet Obstruction (BOO), Bladder Pain Syndrome (BPS), Chronic Pelvic Pain Syndrome (CPPS), Erectile Dysfunction (ED), Interstitial Cystitis (IC), Lower Urinary Tract Symptoms (LUTS), Nocturia, Stress Urinary Incontinence (SUI), and Underactive/Overactive Bladder Syndrome (U/OAB). To allow a comprehensive capture of publications following conference presentations, we included abstracts from 2015 to 2019, considering the variability in publication timelines. Although the mean time to publication post-conference at the EAU is approximately 8.6 months, we extended our review window to three years to account for longer publication processes and to ensure inclusion of studies with extended review periods [31]. This allowed a comprehensive and thorough analysis of a complete set of data available up to that period, ensuring a robust evaluation of trends and discrepancies over a significant duration.

The relevant abstracts were extracted from the ICS website and, for the EAU Congress, from the supplement of the European Journal of Urology. A Medline search (via PubMed.com) was used to check whether these abstracts had been subsequently published as full-text publications in a scientific journal. Only those abstracts were included in the analysis that had the same or very similar title and at least one identical author in both the conference abstract and the full-text publication.

In order to assess the extent of the variance, a previously published discrepancy scoring system was used to assess the consistency between conference abstracts and their full-text publications in research, ranging from 0 to 3 [32]. Briefly, we categorized discrepancies into four grades: Grade 0, where the congress abstract and the published abstract shared identical data, content, and conclusions or had only one differing data point but maintained the same content and conclusion. Grade 1 applied when there were two or more altered data points with unchanged content and conclusion, or unchanged data with one additional content element but the same conclusion. Grade 2 was assigned when data were similar with at most one additional content element and either the same or a modified conclusion. Grade 3 was used when the published paper involved further research beyond the scope of the abstract, with additional data and content, and conclusions that may or may not have differed from those in the abstract. Changes in data points may have included variations in patient numbers, methodologies, materials, or results [32].

To ensure the integrity and impartiality of our sample selection and discrepancy analysis, our study employed a dual-reviewer system where two independent experts in the field systematically applied the established criteria to identify and evaluate abstracts. This process was designed to minimize selection and scoring biases. Together with the well-established discrepancy scoring system, this methodology allows for a consistent and reproducible assessment of discrepancies, providing a robust framework for our analysis.

Finally, an in-depth analysis of the disciplinary origin of these functional urology-related publications was carried out to determine whether gynecology or urology departments play a leading role in research and publication on the topic of functional urology. In this context, authors and institutions of both conference articles and full-text publications were identified via PubMed and/or Google (web search).

## 3. Results

### 3.1. Overview of Analyzed Abstracts and Publication Patterns

During the period from 2015 to 2019, the EAU congresses presented a total of 6759 abstracts, with 546 of these specifically related to functional urology. There was a notable continuous increase in the number of abstracts submitted to the EAU congresses throughout these years. Of the functional urology abstracts at EAU, 288 (53%) were published as full-text papers in scientific journals (Table 1). Over the course of the study period at the EAU Congress, Lower Urinary Tract Symptoms (LUTS) was the dominant topic with 87 out of 288 abstracts (30%), followed by Underactive/Overactive Bladder Syndrome (UAB/OAB) with 61 abstracts (21%), and Interstitial Cystitis/Urinary Incontinence/Stress Urinary Incontinence (I/UI/SUI) with 59 abstracts (20%). BPS/IC accounted for 31 abstracts (11%), and 50 abstracts (18%) involved other topics.

In comparison, the ICS conferences saw the submission of 3246 abstracts in the same timeframe, with 722 of these focusing on functional urology. The ICS congresses experienced a general upward trend in abstract submission as well, though there were annual fluctuations in the numbers. Out of the functional urology-related abstracts at ICS, 411 (57%) succeeded in being published in scientific journals (Table 1). Over the course of the study period at the ICS Congress, S/UI (141 abstracts, 34%) and OAB/UA (120 abstracts, 29%) were the most dominant topics, and LUTS was the topic in 77 abstracts (19%), BPS/IC in 59 abstracts (14%), while 14 abstracts (4%) involved other topics.

In terms of journals where subsequent full-text publication of some of these abstracts occurred, the publication footprint of the congress abstracts from the EAU and ICS is extensive, with a significant portion finding their way into leading scientific journals. Specifically, 122 of the 288 EAU congress abstracts were published in eight prominent journals (*Neurourology & Urodynamics*, *International Urogynecology Journal*, *International Journal of Urology*, *Journal of Urology*, *Urology*, *LUTS*, *European Urology*, *World Journal of Urology*), while the remainder were spread across 66 other journals. For the ICS congress, 220 out of 411 total abstracts were accommodated by the same eight journals, with the rest being distributed among 74 different journals.

### 3.2. Discrepancy Scores Analysis

The systematic content analysis, comparing all abstracts from the EAU and ICS congresses with their subsequent full-text publications, revealed that a majority of abstracts from both congresses exhibited minor to moderate discrepancies upon publication, with the ICS abstracts demonstrating a slightly higher overall agreement with the final publications. Specifically, the analysis highlighted that the lowest discrepancy levels (0 and 1) were most prevalent, indicating a general consistency between the abstracts presented at the congresses and those published in scientific journals (Table 2).

### 3.3. Origin of Publications by Speciality

Out of 699 conference abstracts on functional urology that were subsequently published as full-text articles, urology departments were credited with 446 publications, with 423 being conducted solely by urology departments and 23 resulting from collaborations between urology and gynecology departments. In contrast, gynecology departments were responsible for only 53 independent publications throughout the study period. Additionally, departments from other disciplines, i.e., radiology, oncology, surgery, or pharmacy, contributed to a further 200 abstracts on urogynecology-related topics.

**Figure 1:**
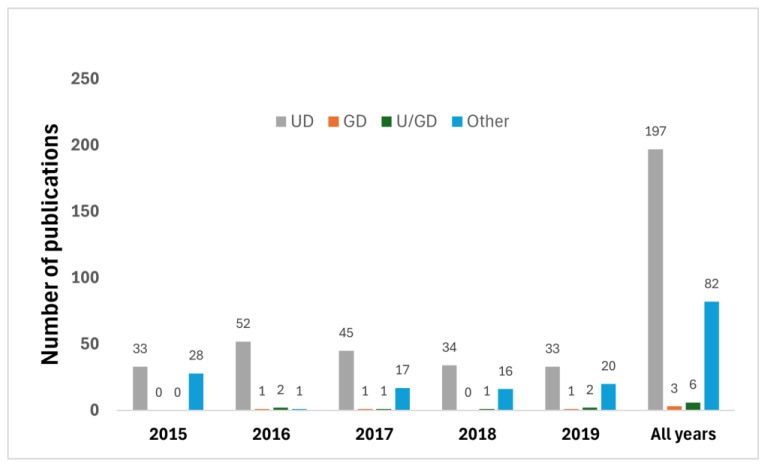
Distribution of studies presented at EAU and published as full-text articles, according to discipline. GD: Gynecology department; UD: Urology department; U/GD: Urology/Gynecology department (cooperation).

During the study period at the EAU congress, urology departments took a leading role in the publication of the 288 abstracts, accounting for 197 abstracts (68%). On the other hand, gynecology’s contribution was minimal, with just three abstracts (1% of the total), with collaborative efforts between gynecology and urology departments making up six abstracts (2%). Other disciplines emerged as the second-largest contributor, with 82 abstracts (28% of publications) (Figure 1).

**Figure 2:**
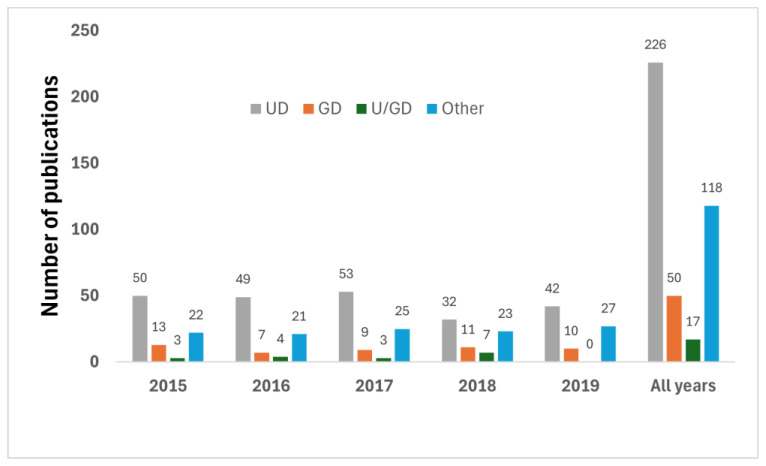
Distribution of studies presented at ICS and published as full-text articles, according to discipline. GD: Gynecology department; UD: Urology department; U/GD: Urology/Gynecology department (cooperation).

In comparison, at the ICS congresses, gynecology’s representation was higher, accounting for 50 of the 411 abstracts (12% of the publications) over the entire period, although still markedly lower than urology’s 226 abstracts (55%). The share of publications resulting from joint efforts between gynecology and urology was marginally higher at the ICS than at the EAU, at 17 abstracts (4%). Other specialties accounted for 118 abstracts (29% of the publications), underscoring a broader interdisciplinary engagement at the ICS compared to the EAU in the field (Figure 2).

## 4. Discussion

The aim of this study was to quantify and analyze the proportion of functional urology abstracts presented at the EAU and ICS Congresses that were later published as full scientific papers, using a bespoke discrepancy index to evaluate differences between conference conclusions and final publications, and to investigate the disciplinary origins of the publications to assess the contributions of various medical specialties to the field.

This study found that over the period from 2015 to 2019, a significant portion of functional urology abstracts presented at the EAU and ICS congresses were published as full-text articles, with the analysis revealing minor to moderate discrepancies between conference abstracts and their published versions. However, the field showed a marked imbalance in the disciplinary origins of publications, with urology departments significantly outweighing gynecology in the production of functional urology-related research.

Our analysis of discrepancies between conference abstracts and full-text publications aligns with similar findings across various medical specialties, confirming that this issue is not confined to urology and gynecology but is prevalent throughout the medical sciences [23]. This similarity across disciplines validates our research methods and underscores the reliability of our results. The consistent presence of these discrepancies highlights the urgent need for continued improvements and rigorous scrutiny within the fields of urology and gynecology. Our study calls for enhanced reporting standards and more stringent peer-review processes to ensure that findings presented at conferences and subsequently published are accurate and truly reflective of current research. Ultimately, by aligning our results with the existing literature, we aim to deepen the understanding of how these discrepancies affect clinical outcomes and advance medical research, emphasizing the need for targeted interventions to improve research transparency and integrity.

Our study evaluates discrepancies between conference abstracts and their subsequent full-text publications in functional urology, uncovering a spectrum of deviations that reflect varying degrees of research evolution and integrity. Grade 0 discrepancies indicate no significant changes, suggesting that the initial findings were robust, reflecting high scientific rigor. Grades 1 and 2 involve minor to moderate adjustments, such as additional data points or slightly altered content, typically in response to peer feedback or further analysis. These changes denote the dynamic nature of ongoing research without undermining its validity. However, Grade 3 discrepancies, which involve significant data expansion or methodology changes, raise concerns about the original study’s design and potentially question the integrity of the research. Such substantial discrepancies can impact clinical decision-making and guideline development, as abstracts are often the first point of interaction with new findings [23,33,34]. This inconsistency necessitates a cautious approach among clinicians, researchers, and policymakers, highlighting the need for rigorous peer review and a critical evaluation of full-text publications [27]. Ultimately, addressing these discrepancies is crucial for ensuring the accuracy and reliability of scientific reporting in functional urology, enhancing the field’s impact on patient care and advancing medical practice.

The present study analyzes the evolution of discrepancy rates between conference abstracts and their full-text publications in urology and gynecology over a five-year period, offering vital insights into the dynamics of scientific reporting. By examining temporal trends, we can understand the impact of evolving methodologies, peer review, and publication standards. Trends in these discrepancies provide empirical evidence for policy makers and academic leaders to develop interventions aimed at enhancing scientific communication’s reliability and integrity. For instance, decreasing discrepancies may indicate effective recent improvements in editorial policies or researcher education, while persistent or increasing discrepancies suggest areas requiring further reform. Overall, this analysis not only aids in understanding discrepancies but also informs strategies to enhance research dissemination and improve clinical outcomes. The analysis of disciplinary contributions within the field of urogynecology highlights a significant dominance of urology over gynecology, a finding that carries implications for both interdisciplinary research and clinical practice. This predominance of urology in the publication of functional urology-related research, as observed in our study, could reflect several underlying factors that shape the landscape of functional urology.

The dominance of urology research may reflect its historical prominence and the early establishment of urology as a specialty focused on surgical interventions for pelvic floor disorders and incontinence [35]. Despite urology departments traditionally receiving higher levels of funding and support, which correlates with increased research productivity [36,37], this imbalance may pose challenges for gynecological research in the field, including difficulties in funding, collaboration, and publication. This discrepancy underscores the need for integrated training and research efforts to include gynecological perspectives, particularly for addressing female-specific conditions, and suggests that enhancing interdisciplinary collaboration could enrich clinical practice in urogynecology, ensuring comprehensive patient care.

Analysis of the congresses’ abstracts and subsequent full-text publications reveals a nuanced picture of interdisciplinary efforts in functional urology. While the dominance of urology over gynecology in the publication records suggests a potential skew in representation, it does not necessarily indicate a lack of collaborative effort in research and clinical practice. Instead, it may highlight the platforms’ roles and their perceived specialties’ focus areas. The EAU Congress, with its strong urology emphasis, and the ICS Congress, known for its broader focus on continence care and pelvic floor dysfunction, serve as crucial venues for sharing research and clinical advancements. However, the publication patterns observed might not fully capture the extent of interdisciplinary work occurring behind the scenes or in clinical settings, where collaborative patient care strategies are developed and implemented.

The interdisciplinary nature of functional urology, underscored by collaborative efforts among urologists, gynecologists, and urogynecologists, is a defining characteristic of this field [16,35,38,39,40]. Such collaboration is essential for addressing the complex conditions within functional urology, which often transcend the boundaries of traditional medical specialties. The findings from the EAU and ICS Congresses provide a unique lens through which to assess the state of interdisciplinary collaboration within this domain. The results from these congresses, while indicating a possible need for more balanced representation, also hint at the potential for more integrated approaches to research and treatment within functional urology. The evolving complexity of conditions treated under this umbrella necessitates a comprehensive approach that leverages the strengths and perspectives of urology, gynecology, and related fields. Previous research has shown that interdisciplinary research teams can offer innovative solutions that a single specialty might not conceive, leading to breakthroughs in treatment modalities, diagnostic tools, and patient care strategies [41,42,43].

Our study’s analysis of discrepancy scores and disciplinary origins highlights key areas for future research in functional urology, emphasizing the need for improved reporting standards, enhanced interdisciplinary research, and balanced contributions across specialties. Encouraging interdisciplinary collaboration and addressing the current dominance of urology are crucial for integrating diverse expertise and ensuring equitable contributions from all relevant fields, including gynecology. Additionally, exploring underrepresented research topics and evaluating the impact of interdisciplinary care on patient outcomes could uncover new insights and enhance patient care. Collectively, these strategies represent a comprehensive approach to overcoming current challenges and fostering advancements in functional urology.

Our study on functional urology, focusing on discrepancies and disciplinary contributions through the lens of the EAU and ICS Congresses, faces limitations including the selected conferences’ scope and the analysis timeframe of 2015 to 2019, which might limit the generalizability and capture of evolving research dynamics. Furthermore, while the data collected from 2015 to 2019 provide valuable insights, it may not fully reflect recent advancements or changes in the field that have occurred post-2019, which could influence the current applicability of our findings. We focused on abstracts from the European Association of Urology (EAU) and the International Continence Society (ICS) Congresses due to their significant influence in the fields of urology and continence care. However, future studies could expand this analysis to include congresses from related specialties such as gynecology to encompass a broader spectrum of interdisciplinary research and collaboration. The bespoke discrepancy index’s subjectivity also introduces challenges in assessing consistency. Despite these limitations, the study’s comprehensive analysis of abstracts from leading conferences and the novel use of a discrepancy index offer significant insights into the field’s research dissemination and highlight the importance of interdisciplinary collaboration for advancing functional urology.

## 5. Conclusions

The study’s findings highlight a significant proportion of functional urology research presented at EAU and ICS congresses achieving publication, yet reveal some discrepancies between abstracts and full texts, and a dominance of urology over gynecology for functional urology-related research. These results underscore the necessity for enhancing reporting standards and fostering interdisciplinary collaboration to address the disparities and improve clinical outcomes in functional urology.

## Figures and Tables

**Table 1:** Functional urology-related abstracts at EAU and ICS congresses between 2015–2019.

	2015		2016		2017		2018		2019		Total	
	EAU	ICS	EAU	ICS	EAU	ICS	EAU	ICS	EAU	ICS	EAU	ICS
FU-related abstracts, N	88	142	100	150	113	159	117	136	128	135	546	722
Abstracts published as full-text paper *n* (%)	61 (69)	88 (62)	56 (56)	81 (54)	64 (57)	90 (57)	51 (44)	73 (54)	56 (44)	79 (59)	288 (53)	411 (57)

EAU: European Association of Urology; FU: Functional Urology; ICS: International Continence Society.

**Table 2:** Discrepancy scores of comparisons between conference abstracts and abstracts from published full-text papers.

Score	0	1	2	3
EAU (*n* = 288)	108 (38%)	73 (25%)	59 (20%)	48 (17%)
ICS (*n* = 411)	201 (49%)	80 (19%)	62 (15%)	68 (17%)

EAU: European Association of Urology; ICS: International Continence Society. Score 0: no significant discrepancies; 1: minor discrepancies; 2: moderate discrepancies; 3: significant discrepancies.

## Data Availability

The original contributions presented in the study are included in the article, further inquiries can be directed to the corresponding author/s.
